# Metformin Synergistically Enhanced the Antitumor Activity of Celecoxib in Human Non-Small Cell Lung Cancer Cells

**DOI:** 10.3389/fphar.2020.01094

**Published:** 2020-07-22

**Authors:** Nini Cao, Yanyan Lu, Jia Liu, Fangfang Cai, Huangru Xu, Jia Chen, Xiangyu Zhang, Zi-Chun Hua, Hongqin Zhuang

**Affiliations:** ^1^The State Key Laboratory of Pharmaceutical Biotechnology, College of Life Sciences, Nanjing University, Nanjing, China; ^2^Changzhou High-Tech Research Institute of Nanjing University and Jiangsu TargetPharma Laboratories Inc., Changzhou, China

**Keywords:** non-small cell lung cancer, p53, extracellular regulated protein kinases, protein kinase B, preclinical

## Abstract

Celecoxib has potential as an effective antineoplastic agent, but it may exhibit side effects. Given the glucose-addicted properties of tumor cells, metformin is recognized for its inhibitory effect on oxidative phosphorylation. In the present study, we aimed to combine low dose of celecoxib with metformin to alleviate the side effects of nonsteroidal anti-inflammatory drugs (NSAIDs) and overcome potential drug resistance. We found that celecoxib combined with metformin obviously suppressed cell migration and proliferation and induced cell apoptosis. Most importantly, *in vivo* experiments revealed the superior antitumor efficacy of combination treatment with a low dosage of celecoxib (25 mg/kg/day) without apparent toxicity. Further study of the underlying mechanism revealed that the two drugs in combination caused ROS aggregation in NSCLC cells, leading to DNA double-strand breaks and increased expression of the tumor suppressor factor p53. Elevated p53 subsequently caused cell cycle arrest and cell proliferation inhibition. The presence of metformin also sensitized NSCLC cells to celecoxib-induced apoptosis by activating caspase-9, -8, -3, and -7, upregulating the pro-apoptotic proteins Bad and Bax, and downregulating the antiapoptotic proteins Bcl-xl and Bcl-2. Moreover, the superior anticancer effect of combined therapy was also due to suppression of Raf-MEK-ERK cascades and PI3K-AKT signaling, which is conducive to overcoming drug resistance. In addition, either celecoxib alone or in combination with metformin suppressed NSCLC cell migration and invasion by inhibiting FAK, N-cadherin, and matrix metalloproteinase-9 activities. Together, our study provided a rational combination strategy with a low dosage of celecoxib and metformin for preclinical cancer application.

## Introduction

Lung cancer is the leading cause of cancer-related death in the world, accounting for 1.6 million deaths each year (Bray et al., 2018; Herbst et al., 2018). Based on its biology, treatment, and prognosis, the World Health Organization (WHO) divides lung cancer into two categories: non-small cell lung cancer (NSCLC) and small cell lung cancer (SCLC). NSCLC accounts for more than 80% of all lung cancer cases. Chemotherapy is still the most dominant and effective treatment, and the use of small molecule tyrosine kinase inhibitors (TKIs) and immunotherapy has led to unprecedented survival benefits in selected patients. However, traditional single drug chemotherapy is often prone to drug resistance and side effects (Gilligan et al., 2007). With the increase in the number of smokers and the aggravation of environmental pollution, the incidence of lung cancer has increased significantly, and the mortality rate is still high. Therefore, continued research into new drugs and combination therapies is required to expand the clinical benefit to a broader patient population and to improve outcomes of NSCLC.

Celecoxib is a selective inhibitor of cyclooxygenase-2 (COX-2) that is clinically used to relieve inflammatory symptoms caused by rheumatoid arthritis. Recent studies have shown that COX-2 is overactivated in a variety of human malignant cancers, and high expression of COX-2 is positively correlated with tumorigenesis, growth, and metastasis (Attiga et al., 2000; Okamoto et al., 2008; Allaj et al., 2013). Therefore, COX-2 inhibitors, including celecoxib, have been used as potential anticancer agents in preclinical trials (Koki and Masferrer, 2002; Quinones and Pierre, 2019). Celecoxib shows excellent anticancer effects in a variety of cancer animal models (Harris et al., 2000; Koki and Masferrer, 2002). At the same time, celecoxib remains the only FDA-approved nonsteroidal anti-inflammatory drug (NSAID) for patients with familial adenomatous polyposis (FAP) (Rayburn et al., 2009). Recent studies have shown that celecoxib is a feasible and clinically active regimen for the prevention and treatment of lung cancer (Lee et al., 2007; Afsharimani et al., 2014). Due to the side effects of NSAIDs, especially cardiotoxicity, most COX-2-specific agents, such as rooxinb (Vioxx^®^) and valdecoxib (Bextra^®^) (as well as etoricoxib and parecoxib) have been discontinued (Rayburn et al., 2009). Inhibition of COX-2 and inhibition of prostaglandins with crucial physiological functions may result in severe gastrointestinal, renal, and cardiovascular side effects (Gong et al., 2012; Gurpinar et al., 2014). In addition, the chemopreventive efficacy of NSAIDs is controversial, and it is unclear whether this deficiency is caused by dose limiting or resistance factors (Allaj et al., 2013). Therefore, the development of a suitable combination of drugs to relieve the side effects of NSAIDs is conducive to boosting the clinical application of celecoxib.

Metformin is the first-line treatment for type 2 diabetes (T2D) mellitus due to its superior safety profile (Pryor and Cabreiro, 2015). Several studies have reported a protective effect against NSCLC in diabetic patients treated with metformin (Libby et al., 2009; Tsai et al., 2014). Aside from the protective effect, metformin can also exert an anticancer effect on patients without metabolic disorders, which was revealed by a randomized phase II study testing in postmenopausal breast cancer patients without insulin resistance and with normal baseline insulin serum levels (Campagnoli et al., 2012). Tumor cells have the characteristic of depleting glucose, while metformin can inhibit oxidative phosphorylation and improve the tumor microenvironment (Ding et al., 2019; Elgendy et al., 2019). In addition to the possibility of a direct antitumor activity of metformin as a single agent in specific contexts, metformin could be used in rational combinations with targeted therapies, in consideration of their limited success. Enhanced antitumor efficacy after combination treatment with metformin has been reported. Shi et al. reported that metformin can enhance lymphoma cell sensitivity to the anticancer agent doxorubicin and mTOR inhibitor temsirolimus (Shi et al., 2012). Storozhuk et al. also found that metformin sensitized NSCLC cells to radiation through the ATM-AMPK-p53/p21 and mTOR-eIF4E axes (Storozhuk et al., 2013). Another report showed that metformin enhanced the response of NSCLC to an EGFR-TKI chemotherapeutic agent (Li et al., 2014).

Based on the superior anticancer effect of celecoxib and the safety profile of metformin, as well as the enhanced sensitivity to celecoxib when combined with metformin, this study verified the anticancer potency of combination therapy with celecoxib and metformin for the first time and explored the mechanisms of tumor inhibition at the cellular level. The experimental results showed that the combination drug treatment has a synergistic inhibitory effect on cell migration, proliferation, and apoptosis, which is attributed to the DNA damage caused by reactive oxygen species (ROS) aggregation, followed by an increase in the expression of the tumor suppressor p53. These tumor inhibitory effects are not only mediated by p53; ERK and PI3K-AKT signaling pathways are also involved. *In vivo* experiments showed that combination therapy inhibits tumor growth in A549 xenograft-bearing nude mice more effectively than metformin and celecoxib alone. This study provides an effective combination treatment strategy for patients with NSCLC.

## Materials and Methods

### Materials

A549 and H1299 cells were purchased from the American Type Culture Collection (ATCC, Philadelphia, PA, USA). Antibodies used for WB are listed as following: β-actin (Abgent, San Diego, USA), N-Cadherin, p-FAK, FAK, MMP-9, Cleaved PARP, Cleaved caspase-9, Cleaved caspase-8, Cleaved caspase-7, Cleaved caspase-3, Bcl-2, Bcl-xl, Bad, Bax, Cyto-c, γ-H2AX, p53, p21, Cdc25A, p-Raf, Raf, p-MEK, MEK, p-ERK, ERK, p-PI3P, PI3P, p-AKT, AKT (Cell Signaling Technology, Beverly, MA, USA), p-ATM, p-CHK2, CDK2 (Abcam Inc., Cambridge, MA). Celecoxib(≥98%,HPLC), metformin(purity: > 99.9%), and pifithrin-α were purchased from Sigma (St. Louis, USA).

### Cell Culture

A549 and H1299 cells were grown in DMEM (Invitrogen, Carlsbad, CA, USA) supplemented with 10% (v/v) fetal bovine serum (FBS; Invitrogen, Carlsbad, CA, USA). All cells were cultured in a humidified CO_2_ incubator at 37°C.

### Cell Viability Assays

Cells were digested and counted by an automated cell counter (Invitrogen, Carlsbad, CA, USA), and 100 μl of 5,000 cells were added to each well in a 96-well plate. Cells were incubated for 12 h and cultured in the incubator to form monolayers. The 96-well plate was changed to cell culture medium with different drug concentrations (metformin: 4 mM, 8 mM, 10 mM, 12 mM, 16 mM; celecoxib: 4 μM, 8 μM, 10 μM, 12 μM, 16 μM), and then incubated for an additional 24 or 48 h. Cell viability was determined by the CCK-8 kit (Beyotime Inst Biotech, China). The absorbance was measured at 450 nm by a TECAN Safire Fluorescence Absorbance and Luminescence Reader (Vienna, VA, USA).

Cells were seeded in a 12-well plate and incubated for 48 h. EdU assay was performed using the BeyoClick™ EdU Cell Proliferation Kit with Alexa Fluor 488 (Beyotime Inst Biotech, China). Briefly, cells were incubated with EdU working solution for 1.5 h. Then, cells were fixed with 4% (v/v) paraformaldehyde for 20 min at room temperature. Next, cells were permeabilized with 0.3% (v/v) Triton X-100 in PBS for 15 min at room temperature after washing with 3% (m/v) BSA PBS solution. Then, the cells were incubated with Click Reaction Buffer for 30 min at room temperature in the dark. Hoechst 33342 was added to each well and incubated for 10 min in the dark at room temperature. Finally, cells were photographed by fluorescence microscope (Zeiss, Jena, Germany).

### Transwell Assay

Cell invasion was detected using transwell chambers (8-μm pore size; Millipore). In brief, 600 μl of complete medium was added to the bottom chamber, and 4 × 10^4^ cells suspended in 200 μl culture media with 10 mM metformin and 25 μM celecoxib alone or in combination were placed in the upper chamber. A cotton swab was used to softly remove cells on the top surface of the membrane after 24 h. The upper chamber was washed twice with PBS and then fixed in 4% (v/v) paraformaldehyde and stained in 0.1% (m/v) crystal violet solution for 30 min. Cells adhering to the bottom surface of the membrane were counted in five randomly selected areas under a 100× microscope field. All data were normalized with a control chamber that contained cells with no treatment.

### Wound Scratch Assay

Cells were plated in 12-well culture plates to form a cell monolayer (near 70% confluence). After serum starvation for 12 h, a wound was made with a sterile P-200 micropipette to scrape off the cells. The wells were then washed three times with PBS to remove nonadherent cells and incubated in medium containing 10% FBS with metformin and/or celecoxib for 24 h. The progress of wound closure was monitored with microphotographs of 10× magnification taken with a light microscope (Carl Zeiss Axioplan 2) at the beginning and end of the experiments after washing with PBS.

### Measurement of ROS

Cells were treated with metformin and/or celecoxib in a 6-well plate for 48 h. Then, cells were incubated with 1 mL 10 μM DCFH-DA (Beyotime Inst Biotech, China) which was diluted with DMEM at 37 °C for 20 min. Cells were washed three times with DMEM before being subjected to flow cytometry analysis (BD Biosciences, CA, USA).

### Mitochondrial Membrane Potential Assay

Detection of mitochondrial membrane potential was performed by Mitochondrial Membrane Potential Assay Kit with JC-1 (Beyotime Inst Biotech, China). NSCLC cells were treated with 10 mM metformin and/or 25 μM celecoxib for 48 h. Cells were digested and collected in cell culture media, and then JC-1 staining solution was added in the dark, and discarded after incubation for 20 to 30 min at 37°C. Cells were washed for three times. Sample was analyzed by flow cytometry (BD Biosciences, CA, USA). Quantification of mitochondrial membrane potential was analyzed by CellQuest software (BD Biosciences, CA, USA).

### Cytotoxicity Assay

Detection of cytotoxicity was performed by LDH Cytotoxicity Assay Kit (Beyotime Inst Biotech, China). Cells were seeded in 96-well plates and treated with 10 mM metformin and/or 25 μM celecoxib for 48 h. 10% (v/v) LDH release reagent was added in each well and cells were incubated for additional 1 h. Supernatant was collected in a new 96-well plate after centrifuging at 400×*g* for 6 min. 33.3% (v/v) LDH test solution was added in supernatant and the supernatant was incubated for 30 min in the dark. The absorbance was measured at 490 nm by TECAN Safire Fluorescence Absorbance and Luminescence Reader (Vienna, VA, USA).

### TUNEL Assay and Immunofluorescence Assay

NSCLC cells were treated with 10 mM metformin and/or 25 μM celecoxib for 48 h. Cells were washed twice with PBS and then fixed in 4% (v/v) paraformaldehyde and permeabilized with 0.2% (v/v) Triton X-100 in PBS for 5 min. The TUNEL assay was performed by using a One Step TUNEL Apoptosis Assay Kit (Beyotime Inst Biotech, China). In brief, TUNEL detection solution was added to each sample and incubated at 37°C for 60 min. Then, the genomic DNA of apoptotic cells was broken, and the exposed 3′-OH was catalyzed by terminal deoxynucleotidyl transferase (TdT) to add dUTP labeled by FITC. After washing with PBS, the cells were restained with propidium iodide (PI). The fluorescent photos of the cells were captured by a fluorescence microscope (Zeiss, Jena, Germany). For immunofluorescence staining, antibodies against γ-H2AX (1:500 dilutions; Cell Signaling Technology, MA, USA) were used and then conjugated with Alexa Fluro^®^ 488 goat anti-rabbit IgG (1:2000 dilutions; Invitrogen, Carlsbad, CA). The fluorescent photos of the cells were captured by a fluorescence microscope (Zeiss, Jena, Germany).

### Cell Cycle Analysis

Cells were seeded in 6-well plates. After 48 h of incubation in full growth media with 10 mM metformin and 25 μM celecoxib alone or in combination, the old medium was collected, and cells were washed twice with PBS before being digested by trypsin. Then, the cells were collected gently with old medium after digestion, washed twice with cold PBS and collected by centrifugation at 1000×*g* for 5 min at 4°C. Ethanol (70%, v/v) was used to fix cells overnight at 4°C. Fixed cells were washed and stained with propidium iodide (PI; 2 mg/mL) in PBS with RNase A (0.1 mg/mL) for 30 min at room temperature in the dark. The distribution of cells with differing DNA content was analyzed on a FACSCalibur flow cytometer with CellQuest software (BD Biosciences, CA, USA) at an excitation wavelength of 530 nm. Fluorescence emission was measured using a 620 nm bandpass filter.

### Detection of Apoptotic Cell Death

Phosphatidylserine on the external surface and propidium iodide uptake were quantified by annexin V-FITC & propidium iodide (PI). Cells were cultured in 6-well plates for 48 h in full growth media in the presence or absence of 10 mM metformin and 25 μM celecoxib alone or in combination. Old medium was removed, and cells were washed twice with cold PBS. Next, cells were collected after digestion with 200 μl trypsin and then softly washed with cold PBS. The cell pellets were resuspended in HEPES buffer and incubated on ice for 20 min with annexin V-FITC (1 mg/mL). Propidium iodide (20 μg/mL) was added before flow cytometry analysis (BD Biosciences, CA, USA). Quantification of apoptotic cells was performed with CellQuest software (BD Biosciences, CA, USA).

### Protein Extraction and Western Blotting

Whole cell lysate was prepared with RIPA buffer (Beyotime Inst Biotech, China) containing protease inhibitors, PMSF and orthovanadate. Total protein was denatured by heating and separated on an SDS-PAGE gel. After transferring to nitrocellulose membrane and blocking with 5% milk in TBS buffer, the protein of interest was immunoplexed with the indicated primary antibody and corresponding secondary antibody. Bound antibodies were then visualized with ECL plus Western blotting detection reagents (GE Healthcare). Signal intensity was quantified by densitometry using Image J software (NIH, Bethesda, MD). All experiments were performed at least three times independently.

### Animals

Athymic nude mice (6–8 weeks of age) were obtained from Shanghai Laboratory Animal Center (Shanghai, China) and housed under germfree conditions. All animals received human care according to Chinese legal requirements. The experiments were approved by Nanjing University Animal Care and Use Committee, and we strictly followed these rules during our experiments.

### *In Vivo* Xenograft Tumor Model of Human NSCLC

A549 cells (5×10^6^ cells in 100 μl) were injected subcutaneously into the dorsal flanks of mice. When the tumor volume reached approximately 100 mm^3^, mice with similar tumor volumes were randomly divided into 4 groups with at least 8 mice in each group. The mice were then gastrointestinal administrated with metformin (150 mg/kg/mouse) once a day, intraperitoneally (i.p.) injected with celecoxib (25 mg/kg/mouse) every two days, or administered with both drugs for a total of three weeks. Control mice were i.p. injected with PBS. The antitumor activity of the treatments was evaluated by assessing tumor growth inhibition. The formula tumor volume = length × width^2^ × 0.52 was used to represent the tumor volume. At the end of the study, the tumors were collected and weighed. Body weight was also examined to evaluate the toxicity of different treatments *in vivo*.

In a parallel animal assay (a total of 4 groups, and 3 mice per group), the tumor establishment and drug treatment were the same as described above. After the mice were euthanized, the tumors were collected, fixed with 4% formaldehyde, and embedded in paraffin. Hematoxylin-eosin (H&E) staining was performed according to standard histological procedures. Apoptotic cells in tumor sections (two sections per mouse, three mice in total) were visualized by the TUNEL technique according to the manufacturer’s instructions (Vazyme, Nanjing, China). Moreover, the paraffin sections (two sections per mouse, three mice in total) of tumors in nude mice were immumohistochemically stained with anti-Cleaved caspase-3 antibody (1:500 dilutions; Cell Signaling Technology, MA, USA) for 2 h. Bound antibody was detected with polymerized HRP anti-rabbit IgG (Maixin, Fuzhou, China) using diaminobenzidine tetrahydrochloride (DAB) as the substrate.

### Statistical Analysis

Statistical analysis was carried out using the SPSS software (version 11.0; SPSS, Chicago, IL). Data were expressed as the mean ± standard deviations (SD). For paired data, statistical analyses were performed using two-tailed Student’s t-tests. For multiple comparisons, statistical analyses were performed using one-way analysis of variance (ANOVA) with a Tukey post-test. For all analyses, *P* < 0.05 was considered statistically significant.

## Results

### Screening of Cell Lines and Combination Conditions

**Figure 1:**
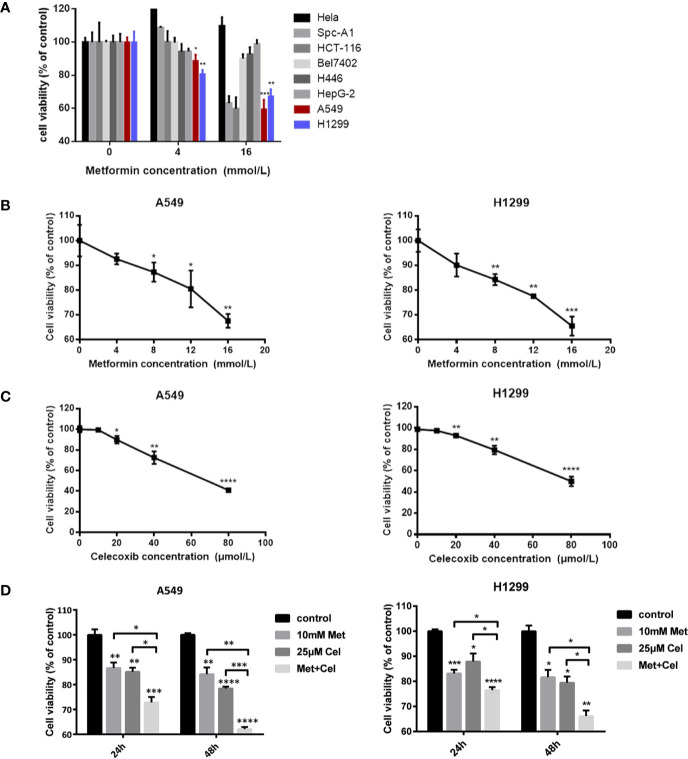
Screening of cell lines and combination conditions. **(A)** Effects of metformin on cell vitality of different cancer cell lines. **(B)** A549 cells and H1299 cells were treated with metformin at different concentrations (0, 4, 8, 12, 16 mM) for 24 h, and the cell viability was assessed by CCK8 assay. **(C)** A549 cells and H1299 cells were treated with celecoxib at different concentrations (0, 10, 20, 40, 80 μM) for 24 h and the cell viability was assessed by CCK8 assay. **(D)** A549 cells and H1299 cells were treated with 10 mM metformin and/or 25 μM celecoxib under different time (24 h or 48 h), and the cell viability was assessed by CCK8 assay. Data are represented as mean ± SD. *p < 0.05, **p < 0.01, ***p < 0.001, ****p < 0.0001.

CCK-8 analysis was performed to determine the suitable cell line and drug concentration that metformin can apply before combining with celecoxib. As shown in **Figure 1A**, different concentrations of metformin have different effects on the cell viability of different cell lines. Low doses of metformin (4 mM) only inhibited the cell viability of some cell lines, such as H446, HEPG-2, A549, and H1299, while high dose of metformin (16 mM) inhibited the cell viability of SPC-A1, HCT-116, A549, and H1299. In general, the cell viability of A549 and H1299 cells in NSCLC decreased the most significantly. Therefore, A549 and H1299 cell lines were selected as the subsequent research objects in our study.

Before combined treatment, A549 and H1299 10cells were treated with a certain concentration gradient of metformin or celecoxib for 24 h. As shown in **Figures 1B, C**, the inhibitory effect of metformin or celecoxib on cell viability was concentration dependent. Higher concentrations of metformin or celecoxib resulted in lower survival rates of A549 and H1299 cells. Excessive use of metformin or celecoxib may increase side effects, which is not conducive to the actual use of drugs. Therefore, low doses of drugs, metformin (10 mM) and celecoxib (25 µM), were then selected as the concentration of combination to further explore their synergistic effects on cell viability. As shown in **Figure 1D**, the inhibition of cell viability by celecoxib combined with metformin was better than celecoxib or metformin alone in A549 and H1299 cells, whether at 24 h or 48 h. With the increase in treatment time, the cell viability of metformin alone did not decrease significantly, while that of celecoxib alone decreased significantly, but the decrease was less than that of combination therapy. These results suggest that the inhibition of cell proliferation can be further strengthened by increasing the combination time. Therefore, in the subsequent study, these doses of metformin (10 mM) and celecoxib (25 µM) and the time of drug action for 48 h were selected for combined treatment.

### Combined Effect of Celecoxib and Metformin on the Growth of NSCLC Cells

**Figure 2:**
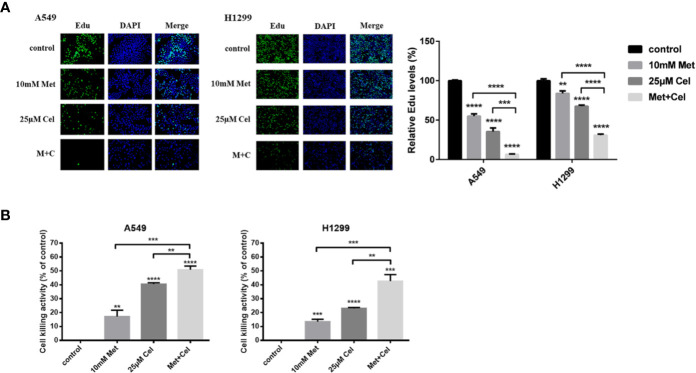
Effect of celecoxib and metformin on the growth of NSCLC cells *in vitro*. **(A)** Detection of DNA proliferation rate in A549 and H1299 cells treated with 10 mM metformin and/or 25 μM celecoxib by Edu. **(B)** Detection of cytotoxicity of A549 and H1299 cells treated with10 mM metformin and/or 25 μM celecoxib by LDH. Data are represented as mean ± SD. **p < 0.01, ***p < 0.001, ****p < 0.0001.

To detect the effect of celecoxib combined with metformin on cell proliferation, DNA proliferation was first detected by EdU fluorescence. After 48 h of drug treatment, EdU was added to detect the proliferation rate of DNA. As shown in **Figure 2A**, compared with the control group, the EdU fluorescence of A549 and H1299 cells treated with metformin or celecoxib alone decreased significantly, suggesting that both drugs could inhibit the synthesis of cell DNA, thereby reducing the cell proliferation rate. Compared with either drug alone, the EdU fluorescence intensity of cells treated with metformin and celecoxib in combination was even lower, indicating that the combined treatment further hindered the synthesis of DNA and significantly decreased the proliferation of cells. Next, cytotoxicity was detected by lactate dehydrogenase release assay (LDH). After 48 h of drug action, the cytotoxic activity was determined by detecting LDH secreted by cells, which indirectly reflected the cell proliferation ability. As shown in **Figure 2B**, celecoxib and metformin can increase cytotoxic activity either alone or in combination. The cytotoxicity of celecoxib was higher than that of metformin in both A549 and H1299 cells. The cytotoxic activity was further increased after the combination of drugs, indicating that the combination of these two drugs inhibited the proliferation of NSCLC cells more significantly.

### Combined Effect of Celecoxib and Metformin on NSCLC Cell Apoptosis

**Figure 3:**
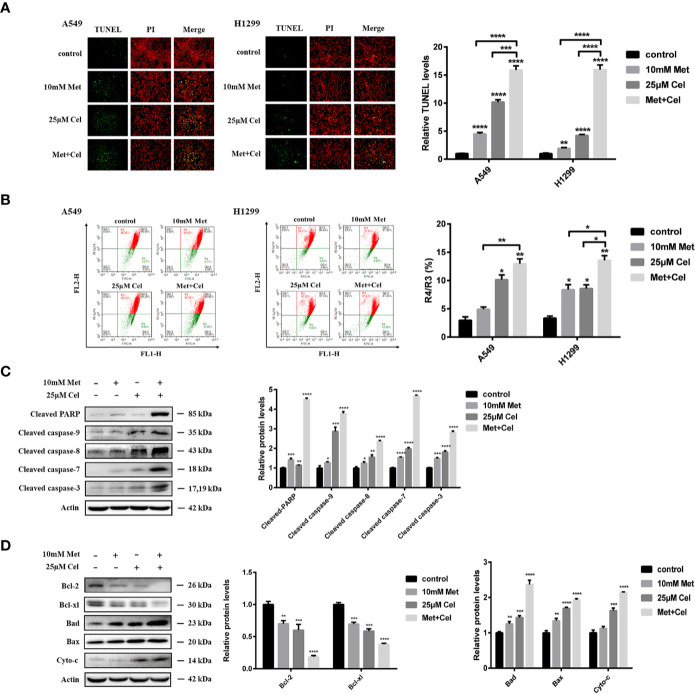
Apoptosis of NSCLC cells induced by celecoxib combined with metformin. **(A)** Detection of apoptosis in A549 and H1299 cells treated with 10 mM metformin and/or 25 μM celecoxib for 48 h by TUNEL assay. **(B)** Detection of MMP in A549 and H1299 cells treated with 10 mM metformin and/or 25 μM celecoxib for 48 h by JC-1. **(C)** Activation of PARP, caspase-9, caspase-8, caspase-7, and caspase-3 was detected by WB analysis in A549 cells under different treatment conditions. All gels run under the same experimental conditions and the representative images of three different experiments were cropped and shown. Band intensity was quantified by Image J software. **(D)** Expressions of the Bcl-2 family proteins, Bcl-2, Bcl-xl, Bad, Bax, and Cyto-c in A549 cells under different treatment conditions were detected by WB analysis. All gels run under the same experimental conditions and the representative images of three different experiments were cropped and shown. Band intensity was quantified by Image J software. Data are represented as mean ± SD. *p < 0.05, **p < 0.01, ***p < 0.001, ****p < 0.0001.

To determine whether tumor cellular viability decreased with metformin and celecoxib *via* apoptosis, we next performed a TUNEL assay. Though the TUNEL fluorescence of cells treated with metformin or celecoxib alone increased, the fluorescence intensity of TUNEL further increased in both A549 and H1299 cells when celecoxib and metformin were used in combination (**Figure 3A**). When cell apoptosis occurs in the early stage, the mitochondrial membrane potential (MMP) decreases; thus, MMP can reflect early apoptosis to a certain extent. After 48 h of drug treatment, JC-1 was added to detect MMP. We observed that MMP was decreased significantly in A549 and H1299 cells treated with celecoxib and metformin in combination compared to those treated with either drug alone (**Figure 3B**), which indicated that celecoxib combined with metformin can promote the early apoptosis of A549 and H1299 cells.

To further explore the mechanisms of metformin and celecoxib-induced apoptosis in NSCLC cells, the activation of caspase family proteins was detected by Western blotting analysis. We found that metformin alone did not activate the caspase family proteins significantly, while celecoxib alone slightly upregulated the levels of cleaved caspase-9, cleaved caspase-8, cleaved caspase-7, and cleaved caspase-3 (**Figure 3C**). However, the expression of cleaved caspase-9, cleaved caspase-8, cleaved caspase-7, and cleaved caspase-3 increased significantly in cells receiving combination treatment (**Figure 3C**). In addition, metformin or celecoxib alone only caused a small amount of PARP cleavage, and cleaved PARP expression was significantly increased after the combination of celecoxib and metformin (**Figure 3C**). Next, we studied the effects of celecoxib and metformin on anti-apoptotic (Bcl-xl and Bcl-2) proteins and pro-apoptotic (Bad and Bax) proteins of the Bcl-2 family in A549 cells. The results showed that both drugs alone or in combination decreased the expression of Bcl-xl and Bcl-2 and increased the expression of Bad and Bax, especially in the combined group (**Figure 3D**). These data suggest that celecoxib combined with metformin can activate both exogenous and endogenous apoptosis signaling pathways to induce apoptosis in NSCLC cells.

### The Combination of Metformin and Celecoxib Increases ROS and DNA Damage in NSCLC Cells

**Figure 4:**
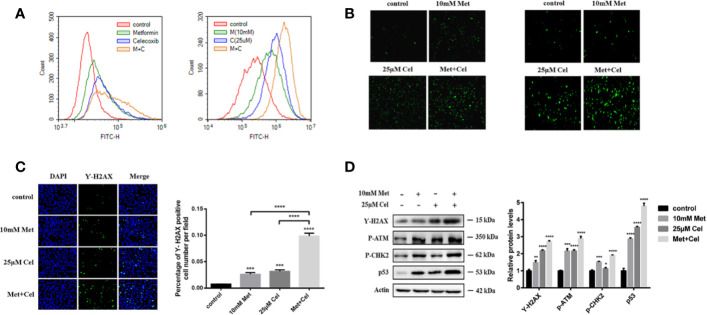
Combined treatment with celecoxib and metformin increases reactive oxygen species and DNA damage in NSCLC cells. **(A)** Detection of ROS accumulation in A549 and H1299 cells by fluorescent probe DCFH-DA. The change of fluorescence intensity was observed by flow Cytometry. **(B)** Detection of ROS accumulation in A549 and H1299 cells by fluorescent probe DCFH-DA. The change of fluorescence intensity was observed by Fluorescence microscope. **(C)** Immunofluorescence staining of γ-H2AX in A549 cells treated with 10 mM metformin and/or 25 μM celecoxib. **(D)** Effects of celecoxib and metformin on γ-H2AX, p-ATM, p-CHK2, and p53 protein expression. A549 cells were treated with 10 mM metformin and/or 25 μM celecoxib for 48 h, and then harvested for WB analysis using indicated antibodies. The level of actin served as the loading control. Band intensities were calculated using software Image J. Relative intensities are also shown. Data represent mean values of triplicate samples. Data are represented as mean ± SD. *p < 0.05, **p < 0.01, ***p < 0.001, ****p < 0.0001.

To further explore the changes in intracellular metabolism, ROS were detected in A549 and H1299 cells after 48 h of treatment with celecoxib and metformin alone or in combination. We observed that either drug alone or in combination could increase the green fluorescence of A549 and H1299 cells (**Figures 4A, B**), indicating that the level of intracellular ROS increased after drug treatment. Compared with single drug treatment, celecoxib combined with metformin further enhanced the green fluorescence (**Figures 4A, B**), indicating that the combination of metformin and celecoxib significantly increased the level of ROS in cells. Excessive active oxygen can induce DNA damage and double-strand breaks (DSBs) of DNA. γ-H2AX is a marker of DSBs. DSB of DNA was then detected by immunofluorescence. We observed that either drug alone or in combination could enhance the fluorescence of γ-H2AX in A549 cells (**Figure 4C**), indicating that both drugs could cause DSBs. Compared with the single drug alone, the expression level of γ-H2AX increased significantly in cells receiving combined treatment (**Figure 4C**), indicating that celecoxib combined with metformin can increase DNA damage in A549 cells. To further test the DNA damage of cells, changes in DNA damage-related proteins were also detected by immunoblotting assay. The expression level of γ-H2AX was consistent with that of immunofluorescence (**Figure 4D**), indicating that celecoxib combined with metformin can facilitate DSBs and cause injury. DNA injury can upregulate the expression of p-ATM, p-CHK2, and p53. In our study, though either drug alone or in combination upregulated the expression of p-ATM, p-CHK2, and p53, the upregulation of p-ATM, p-CHK2, and p53 was the most significant in the combined treatment group (**Figure 4D**), which indicated that the combination of these two drugs could activate the signal pathway of DNA damage better than that of either drug alone.

### Combined Celecoxib and Metformin Treatment Arrests the Cell Cycle at the G1 Phase Through Regulation of p53

**Figure 5:**
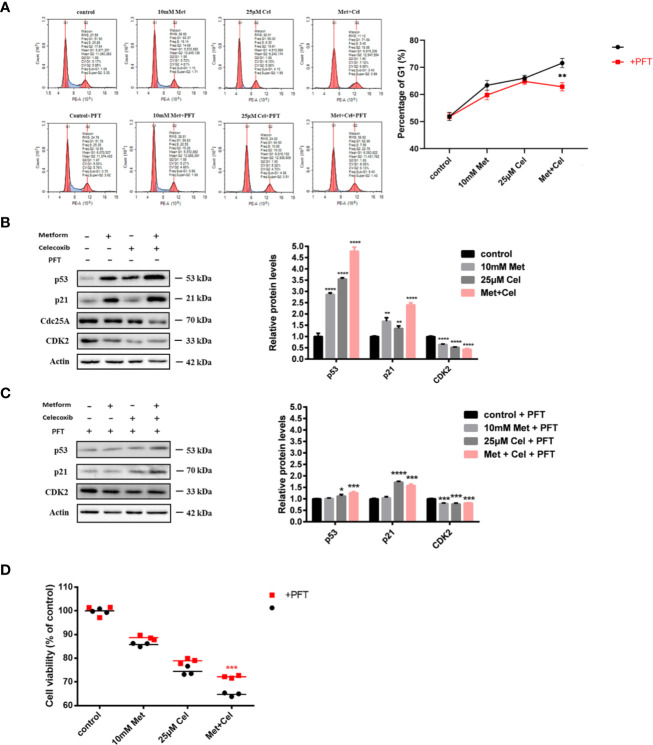
Combined treatment with celecoxib and metformin acts on p53 to arrest cell cycle in the G1 phase and enhance antiproliferative response. **(A)** Cell cycle analysis of A549 treated with 10 mM metformin and/or 25 μM celecoxib for 48 h with the absence or presence of 5 μM PFT. Quantification of the proportion of the cells in the G1 phase was shown in the right panel. **(B)** Western blotting analysis of p53, p21, Cdc25A, CDK2 protein levels of A549 cells treated with 10 mM metformin and/or 25 μM celecoxib for 48 h. Band intensity was quantified by Image J software. **(C)** Western blot analysis of p53, p21, Cdc25A, CDK2 protein levels of A549 cells treated with 10 mM metformin and/or 25 μM celecoxib for 48 h in the presence of 5 μM PFT. Band intensity was quantified by Image J software. **(D)** Cell viability analysis of A549 cells treated with 10 mM metformin and/or 25 μM celecoxib for 48 h in the absence or presence of 5 μM PFT. Data are represented as mean ± SD. *p < 0.05, **p < 0.01, ***p < 0.001, ****p < 0.0001.

Celecoxib has been reported to have the ability to inhibit cell proliferation to execute its antitumor effect. Therefore, we next detected the effect of celecoxib, metformin or their combination on cell cycle distribution. As shown in **Figure 5A** upper panel, both metformin and celecoxib treatment alone could cause arrest of the cell cycle at the G1 phase, while arrest at the G1 phase was more obvious under combination drug treatment, and the number of cells increased by nearly 20% compared with the control group without any drug treatment. The tumor suppressor p53 is critical for the DNA damage-induced checkpoint, which coordinates cell cycle progression with DNA repair. To explore whether cell cycle arrest caused by combination drug treatment was mediated *via* p53, a p53 inhibitor (pifithrin-α, PFT) was then used. As shown in the low panel of **Figure 5A**, the addition of PFT slightly alleviated G1 phase arrest in cells treated with either drug alone; however, there was a significant decrease in the proportion of cells in G1 phase in the combination group, which reveals that combination treatment with celecoxib and metformin might arrest the cell cycle in G1 phase through the p53 pathway. In addition, the changes in proteins related to p53 and G1/S regulation were further examined by Western blotting analysis in the presence or absence of PFT. As shown in **Figure 5B**, in the absence of PFT, a single drug alone or the combination treatment upregulated the protein levels of p53 and p21. Moreover, the expression of Cdc2 and CDK2 decreased after the combination treatment. The effect of this regulation was the most significant in the combination group, which was consistent with the results of the cell cycle analysis. However, after the addition of PFT, there was relatively less upregulation of p53 and its downstream target p21 in the combined group, and the downregulation of CDK2 was also attennuated (**Figure 5C**), indicating that PFT can inhibit the drug combination-induced protein changes related to the G1 phase. These results solidified the previous conclusion that the combination of metformin and celecoxib blocked the cell cycle in G1 phase through the p53 pathway.

Due to the correlation between G1 phase arrest and anti-proliferation, further experiments were performed to explore whether the anti-proliferation effect in the combination group was also mediated *via* p53 using the CCK-8 assay. Compared with the group without the addition of PFT, the group cotreated with PFT exhibited restored cell viability to some extent, and the reversal effect was the most obvious in the combination group, indicating that metformin combined with celecoxib enhanced antiproliferative response through the p53 pathway (**Figure 5D**).

### Celecoxib and Metformin Combined Therapy Induces Apoptosis by Inhibiting the ERK and PI3K/AKT Signaling Pathways

**Figure 6:**
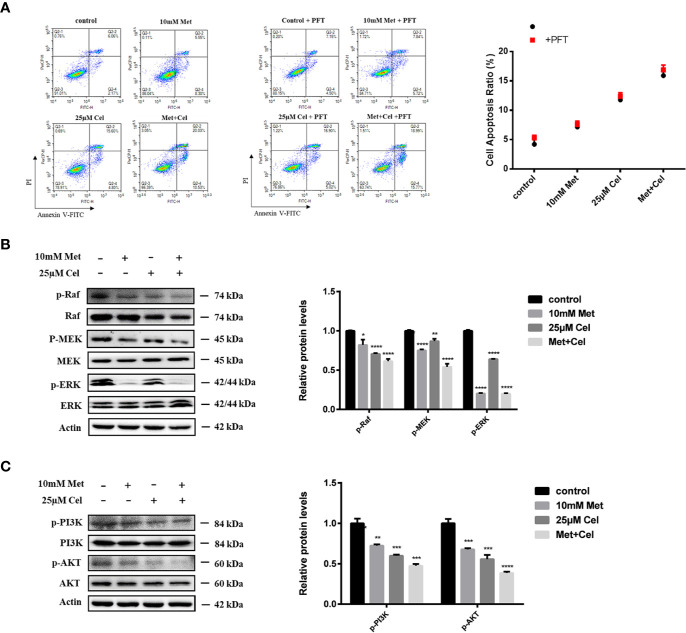
Combined treatment with metformin and celecoxib may induce apoptosis by inhibiting ERK and PI3K/AKT signaling pathways. **(A)** Cell apoptosis analysis of A549 cells treated with 10 mM metformin and/or 25 μM celecoxib for 48 h in the absence or presence of 5 μM PFT. Quantification of the proportion of the apoptotic cells was shown in the right panel. **(B)** Western blot analysis was used to detect the phosphorylation state of Raf, MEK, ERK after treatment with 10 mM metformin and/or 25 μM celecoxib for 48 h. Band intensity was quantified by Image J software. **(C)** Western blot analysis was used to detect the phosphorylation state of PI3K and AKT after treatment with 10 mM metformin and/or 25 μM celecoxib for 48 h. Band intensity was quantified by Image J software. Data are represented as mean ± SD. *p < 0.05, **p < 0.01, ***p < 0.001, ****p < 0.0001.

Combined treatment with the two drugs upregulated p53 protein expression in A549 cells. Then, to examine whether apoptosis induced by combination treatment was also related to upregulated p53, we further detected the apoptotic state of A549 cells with or without PFT addition. As shown in **Figure 6A**, metformin or celecoxib treatment alone led to less than 10% apoptosis, and the ratio of apoptotic cells increased significantly in the combination group, which is consistent with the previous TUNEL and MMP results. In addition, we found that there were no significant changes in the apoptosis rate of the cells in the single or combined drug treatment groups after the addition of PFT, indicating that the apoptosis of A549 cells induced by metformin combined with celecoxib was not mediated by p53.

Previous results showed that the protein levels of Bad, a proapoptotic member of the Bcl-2 family, increased and the expression of Bcl-2 and Bcl-xl diminished in the drug treatment group, particularly in the combination group. We further investigated the changes in related proteins involved in ERK signaling and the PI3K/AKT axis, which are upstream signaling pathways of Bad. The data showed that metformin or celecoxib alone can slightly inhibit the phosphorylation of Raf, MEK, PI3K, and AKT. However, combined treatment with two drugs markedly enhanced this inhibitory effect. Interestingly, we noticed that celecoxib alone had no apparent effect on the level of p-ERK, while metformin alone could suppress the phosphorylation of ERK, indicating its specific role in ERK signaling. Based on these results, we thus hypothesized that metformin combined with celecoxib might act on ERK signaling and the PI3K/AKT axis to regulate Bad protein expression (**Figures 6B, C**).

### The Combined Effect of Celecoxib and Metformin on the Migration of NSCLC Cells

**Figure 7:**
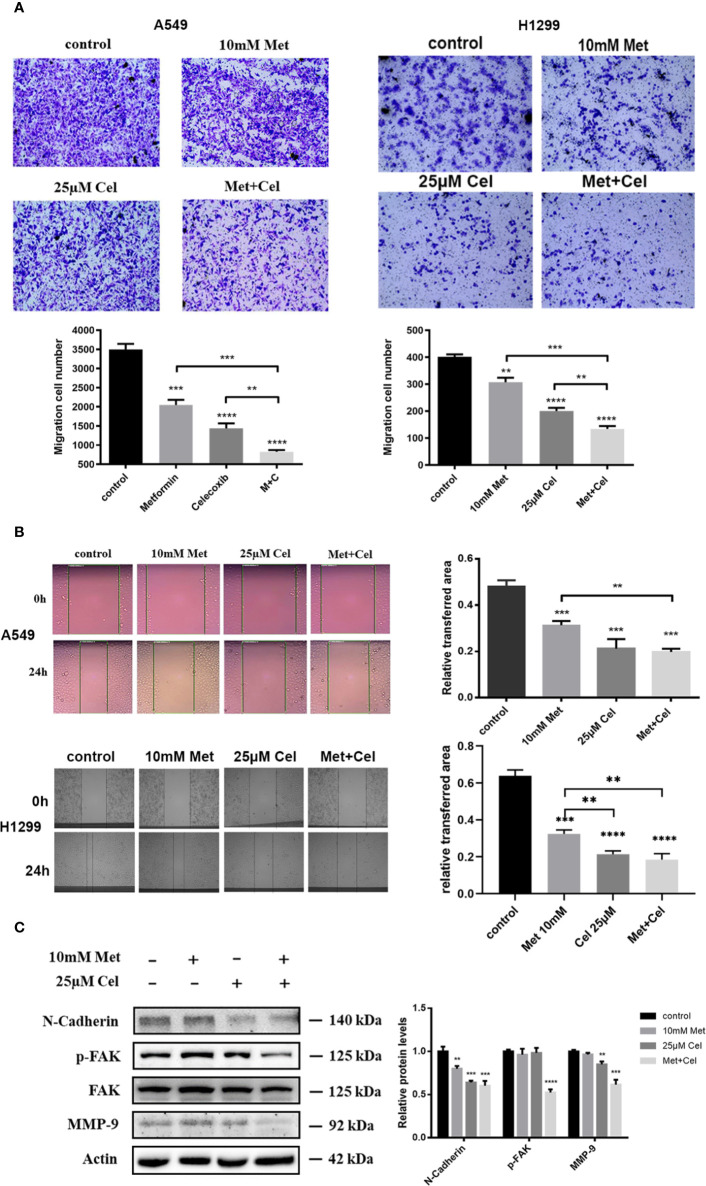
Effect of celecoxib and metformin in combination on NSCLC cell migration. **(A)** Transwell assay. A549 and H1299 cells were treated with 10 mM metformin and/or 25 μM celecoxib. After 12 h pretreatment and 12 h incubation in the upper chamber, the cells migrating to the lower membrane were stained and counted in five fields with a magnification of × 100. The experiments were carried out in triplicate and representative data are shown. **(B)** Wound healing assay. A549 and H1299 cells were treated with 10 mM metformin and/or 25 μM celecoxib. Photographs were taken immediately and after 24 h of creating the scratch. Images shown are representative of three independent experiments. **(C)** Effects of metformin and celecoxib on N-Cadherin, FAK, p-FAK, and MMP-9 protein expression. A549 cells were treated with 10 mM metformin and/or 25 μM celecoxib for 48 h, and then harvested for Western blotting analysis using indicated antibodies. The level of actin served as the loading control. Band intensities were calculated using software Image J. Relative intensities are also shown. Data represent mean values of triplicate samples. Data are represented as mean ± SD. *p < 0.05, **p < 0.01, ***p < 0.001, ****p < 0.0001.

The effects of celecoxib combined with metformin on the invasion and migration of NSCLC cells were further investigated by transwell and scratch experiments. Either drug alone only partially reduced the number of A549 and H1299 cells passing through the migration chamber, while the combination of celecoxib and metformin significantly inhibited the passage of A549 and H1299 cells through the chamber (**Figure 7A**), indicating that the invasive ability of cells decreased significantly in response to celecoxib combined with metformin. In addition, we also noticed that the area of scratch wound healing of A549 and H1299 cells was significantly reduced (**Figure 7B**) when celecoxib was combined with metformin, indicating that the migration ability of the cells was suppressed.

Several proteins, such as N-cadherin, FAK, and MMP-9, were reported to play important roles in cell migration and metastatic potential. Western blotting analysis showed that metformin and celecoxib in combination could significantly reduce the expression of N-cadherin protein and the level of FAK phosphorylation in NSCLC cells; however, the total FAK protein remained unchanged (**Figure 7C**). In contrast, metformin or celecoxib alone had no significant effect on FAK phosphorylation. Compared with celecoxib or metformin single treatment, the expression of MMP-9 in cells treated with celecoxib and metformin in combination markedly decreased (**Figure 7C**). In general, these results suggest that celecoxib combined with metformin can antagonize the activation of N-cadherin, p-FAK, and MMP-9 in A549 cells, thus better inhibiting cell migration and invasion.

### The Combination of Celecoxib and Metformin Suppresses the Development of Lung Cancer Xenografts in Nude Mice

**Figure 8:**
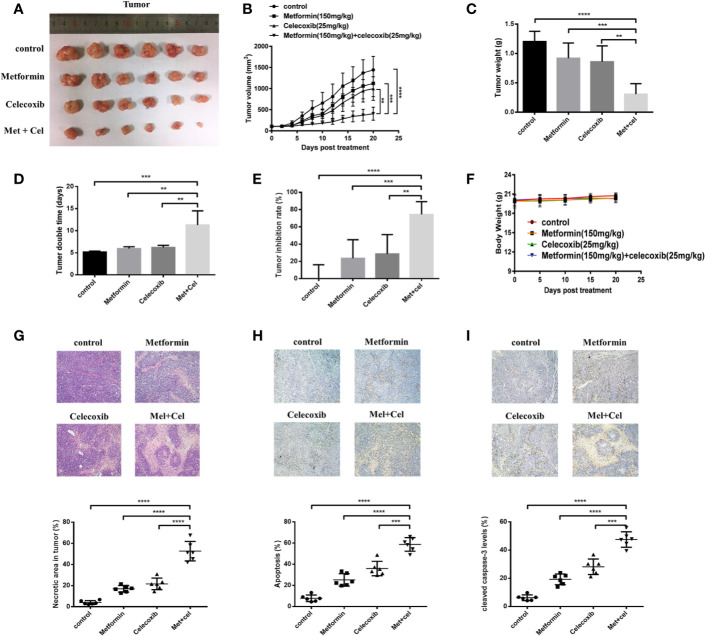
Combined treatment with celecoxib and metformin inhibits tumor growth in A549 tumor-bearing nude mice. A549 cells were injected subcutaneously into the dorsal flanks of athymic nude mice. When tumors reached a size of approximately 100 mm^3^, mice were gastrointestinal administrated with metformin (150 mg/kg/mouse) or intraperitoneally (i.p.) injected with celecoxib (25 mg/kg/mouse) or the combination of two drugs for a total of 20 days. **(A)** Representative images of the excised tumors from each therapeutic group were shown. **(B)** The tumor growth inhibitory effects of different treatments were compared. **(C)** At the end of the study, the excised tumors from each group were weighed. **(D)** Tumor doubling time of each therapeutic group. **(E)** Tumor inhibition rate of each therapeutic group. **(F)** Body weight change during treatment. **(G)** The necrosis changes of tumors receiving different treatments were observed by H&E staining. **(H)** TUNEL staining was applied to detect the apoptotic state of tumors receiving different treatments. **(I)** The amounts of cleaved caspase-3 were measured by immunohistochemistry. Quantification was followed by Image-J. Data are represented as mean ± SD. **p < 0.01, ***p < 0.001, ****p < 0.0001.

To evaluate the *in vivo* efficacy of the drug combination treatment with celecoxib and metformin on NSCLC, nude mice were subcutaneously inoculated with A549 cells. Celecoxib and metformin were administered alone or in combination for 20 days when the tumor grew to approximately 100 mm^3^. The results are shown in **Figure 8**. We found that the tumor volume after combination treatment for 20 days was obviously smaller than that after single drug treatment with metform15in or celecoxib, suggesting that celecoxib combined with metformin therapy has excellent tumor inhibition efficacy when compared with single drug therapy (**Figures 8A, B**). Moreover, the tumor weight of the combination group was significantly lighter than that of the metformin or celecoxib monotherapy group (**Figure 8C**). Analysis of tumor doubling time and tumor inhibition rate showed that the tumor doubling time of the combination group was significantly elongated, nearly twice that of the control group and monotherapy group (**Figure 8D**), and the tumor inhibition rate reached nearly 75% (**Figure 8E**). In addition, there was no significant change in the body weight of nude mice throughout the whole therapy progress (**Figure 8F**), indicating that metformin or celecoxib at this dose had no significant toxic side effects in nude mice.

HE staining was used to detect necrosis of tumor tissue. The results showed that combination therapy contributed to more serious tumor necrosis due to the increased necrotic area in the combination group compared with the monotherapy group (**Figure 8G**). To observe the apoptosis of tumor tissue, paraffin sections of tumor tissue were followed by TUNEL staining and immunohistochemistry. We observed that the apoptotic area was more obvious in the combination group than in the single drug treatment group according to the TUNEL staining results (**Figure 8H**). Further immunohistochemical staining with anti-cleaved caspase-3 antibody also revealed that the expression of cleaved caspase-3 was upregulated after combination therapy, which was more significant than the single drug therapy group (**Figure 8I**). Taken together, the results indicated that celecoxib combined with metformin can increase tumor tissue apoptosis *in vivo*, thus suppressing the development of lung cancer xenografts.

## Discussion

Lung cancer remains the leading cause of cancer-related mortality worldwide, and NSCLC is the predominant type of lung cancer. With the drug discovery and disease diagnosis approaches, an increasing number of targeted drugs are being applied in NSCLC. However, the presence of multiple resistance factors, such as intrinsic resistance, adaptive resistance and acquired resistance, results in a temporary response to the targeted drug. Clinical trials of combination therapy with mainstream targeted agents on NSCLC, such as epidermal growth factor receptor tyrosine kinase inhibitors (EGFR-TKIs) and anaplastic lymphoma kinase tyrosine kinase inhibitors (ALK-TKIs) were also suspended due to the limited therapeutic effects of the drugs (Rotow and Bivona, 2017). Therefore, it is important to find an effective treatment with low toxicity and few side effects that can be applied to NSCLC.

A review has shown the potential and rationale for COX-2 inhibitors in lung cancer (Krysan et al., 2006). Moreover, the efficacy profile of NSAIDs for treating NSCLC has been reported in clinical trials (Chen et al., 2014; Hou et al., 2016). Celecoxib is beneficial in the treatment of advanced cancers but with an increased risk of cardiovascular events (Chen et al., 2014). Given the possible side effects of high dose NSAIDs which may lead to the failure of clinical trials, we expected to use a lower dose of celecoxib in this research. Tai et al. found that high concentrations of celecoxib from 50 μM to 70 μM can significantly cause G1 phase arrest in two liver cancer cells, Bel7402 cells and HepG2 cells (Tai et al., 2019). In this study, a low dose of 25 μM celecoxib in combination with metformin significantly induced cell cycle arrest. At the same time, *in vivo* experiments also demonstrated the superiority of celecoxib combined with metformin in tumor treatment. Tsutsumi et al. showed that 100 mg/kg celecoxib can significantly inhibit tumor growth in MKN-45 cell xenografted tumor nude mice (Tsutsumi et al., 2006). Another study executed on mice bearing A549 tumors by Zhang et al. also displayed superior antitumor effects through oral administration of celecoxib at a dosage of 200 mg/kg/day (Zhang et al., 2017), which is eight times that of 25 mg/kg/day, the dosage that was used in this current study. Generally, a lower dosage of celecoxib was applied in our study, which implies a lower risk of toxic effects in future clinical research.

The occurrence and development of tumors is the result of tumor cell proliferation, apoptosis, and migration, which are affected by multiple conditions. The antiproliferation, proapoptosis, and antimigration effects of celecoxib have been reported in a variety of tumor cells (Jendrossek, 2013; Tai et al., 2019; Tian et al., 2019). Metformin has also been reported to slightly inhibit tumor cell growth (Cheng et al., 2019; Lee et al., 2019), which is consistent with the data in this study. Our study demonstrates that celecoxib combined with metformin can effectively inhibit the proliferation of A549 cells. Next, we explored the mechanism by which celecoxib combined with metformin inhibits cancer cell proliferation and induces apoptosis and cell cycle arrest.

Celecoxib combined with metformin activates the extrinsic and intrinsic apoptosis signaling pathways. The inherent pathway of apoptosis is often unregulated in cancer. Our data suggested that celecoxib combined with metformin activates caspase family proteins which are necessary for the morphological and biochemical characteristics of apoptosis (Mrozik et al., 2018). There are three subfamilies of Bcl-2 proteins: anti-apoptotic Bcl-2 proteins (such as Bcl-2 or Bcl-xl), pro-apoptotic BH3-only proteins (including PUMA (also known as BBC3), Bid and Bim (also known as Bcl2L11)) and pro-apoptotic effector proteins (Bax, Bak, and Bok) (Vandooren et al., 2013). Here, the combined use of drugs further decreased the expression of Bcl-xl and Bcl-2, and increased the expression of Bad and Bax, suggesting that metformin combined with celecoxib also activated the endogenous apoptosis signaling pathway.

In addition, our data suggest that metformin combined with celecoxib led to significant G1 arrest, and this blockade is due to ROS accumulation after drug treatment. ROS are toxic to the cell which results in oxidation of proteins, lipids, and DNA (Vanderauwera et al., 2011). ROS-derived DNA oxidation damages the normal functional bases and sugar residues, leading to DNA single- and double-strand breaks (Amor et al., 1998), (Roldan-Arjona and Ariza, 2009). γ-H2AX, the phosphorylated form of histone H2AX, is a marker of DNA damage, which accumulates near the DNA break site and is rapidly phosphorylated by members of the phosphatidylinositol 3-kinase-associated kinase family, including ATM, ATR, and DNA-activated protein kinases, when DSBs occur (Sone et al., 2014). The DNA damage response (DDR) follows the DSB. The transducer kinases in the DDR are activated and phosphorylate a subset of effectors such as p53, Chk1, and Chk2, that are involved in the initiation of cell cycle checkpoints and apoptosis (Hammond et al., 2007). Our results showed that there is a significant increase in activated ATM in the combination group which results in the accumulation of phosphorylated H2AX followed by the upregulation of p53 and phosphorylated CHK2, which is the subset effector in DDR.

The transcription factor p53 is a vital tumor suppressor that is associated with a wide range of functional genes regulating cell cycle arrest and apoptosis in response to DNA damage (Khoo et al., 2014). Next, we investigated the regulatory effect of p53 on the antimigration, antiproliferation, and proapoptosis effects induced by drug treatment. The results of the cell cycle and apoptosis assays revealed that p53 is a major mediator in cell cycle arrest during the combination treatment with celecoxib and metformin, while the apoptosis caused by the combination treatment is not mediated through p53. Kracikova et al. proved that cells expressing a high level of p53 to overcome the apoptotic threshold, which is determined by the expression level of p53 and its downstream targets in the cellular context (Kracikova et al., 2013), can trigger the apoptosis cascade. Moreover, mutant p53^E177R^ abolishes apoptotic functions due to its inability to bind to DNA caused by the altered quaternary structure of the p53 tetramer (Schlereth et al., 2010; Timofeev et al., 2013). Our results showed that p53 does not mediate the regulation of apoptosis by either drug alone or by the combined treatment, which is inconsistent with previous reports. Therefore, whether elevated p53 is still below the apoptotic threshold or whether a functional mutation in p53 occurs in A549 cells leading to the results obtained in our research needs to be further explored.

Due to the limited regulatory effect of p53, we assumed that the superior antineoplastic efficacy of combination treatment might be due to multitarget effects. Mitogen-activated protein kinase (MAPK) cascades are an evolutionarily conserved signaling pathway in which ERK is a downstream module that is activated by the MAPK/ERK kinase (MEK)1/2 dual-specificity protein kinases regulated by Raf serine/threonine kinases (Roberts and Der, 2007). Our results indicate that both metformin and celecoxib can act on Raf-MEK-ERK cascades. Metformin or celecoxib alone can inhibit the phosphorylation of Raf, MEK, and ERK, and metformin inhibits ERK phosphorylation more thoroughly, which increases the potency of combination treatment with celecoxib. The RAS-RAF-MEK-ERK pathway has been reported to be engaged in the regulation of normal cell proliferation, survival, and differentiation (Samatar and Poulikakos, 2014). We hypothesize that the apoptosis caused by single or combination treatment might be due to the inhibition of ERK signaling. Moreover, the inhibition of ERK signaling may also contribute to the anti proliferative potency due to only partial reversion of A549 vitality after the inhibition of p53 signaling with PFT. Many cancers exhibit deregulated activation of ERK1/2 signaling and an enhanced dependency on ERK1/2 signaling (Caunt et al., 2015). Many inhibitors targeting ERK signaling have been exploited. The level of phosphorylated ERK is usually monitored to reflect the efficacy of RAF or MEK inhibitors on cancer therapy targeting RAS-ERK signaling, and near-complete and long-lasting inhibition of ERK signaling is a reliable guarantee of durable responses (Samatar and Poulikakos, 2014). Since the superior inhibition efficacy of phosphorylated ERK by metformin and this inhibitory effect have been reported by many studies (Wu et al., 2013; Chai et al., 2015; Peng et al., 2016; Yue et al., 2019), it might suggest a potential strategy to help overcome the resistance of ERK signaling inhibitors. We believe that further study on how metformin inhibits ERK signaling without causing unacceptable toxicities can help overcome the challenges in the clinical application of ERK signaling inhibitors.

The signaling network defined by phosphoinositide 3-kinase (PI3K), AKT, and mammalian target of rapamycin (mTOR) controls most hallmarks of cancer, including cell cycle progression, survival, metabolism, motility, and genomic instability (Engelman, 2009; Pal and Mandal, 2012; Fruman and Rommel, 2014). We also investigated the inhibitory effects of combination therapy with celecoxib and metformin on this crucial antineoplastic pathway. Our data revealed that both metformin and celecoxib inhibited PI3K/AKT signaling, among which celecoxib had a better inhibition efficacy than metformin. Combination treatment barely abolished the phosphorylation of AKT. Fruman et al. claimed that targeting the RAS-RAF-MEK-ERK cascade combined with PI3K-AKT-mTOR inhibitors is an attractive strategy for antitumor therapy (Fruman and Rommel, 2014). Both networks can promote cell proliferation and survival, and there is extensive crosstalk between the pathways. Our assumption that the efficient antitumor effect of celecoxib combined with metformin is due to the inhibition of multiple key pathways at the same time was thus proven.

Finally, we observed the effect of the drug combination on cell migration. According to our research, compared with the drug alone, two drugs in combination showed a higher inhibitory effect on migration, indicating that celecoxib combined with metformin can enhance the antitumor migration ability. In many types of solid tumors, the abnormal expression of the cell adhesion molecule N-cadherin is a sign of the transformation of epithelial cells into stromal cells, leading to the invasive phenotype of the tumor. This shift gives tumor cells the ability to escape from the limitations of the primary tumor and transfer to secondary sites (Taylor et al., 2008). Celecoxib plays a major role in inhibiting N-cadherin expression as compared to metformin. Integrin receptor-mediated cell migration accounts for a large part of cell migration. FAK has been proven to be a key regulator of integrin-mediated cell movement, which is phosphorylated when it is activated. In our study, single treatment with metformin or celecoxib had no significant effect on FAK phosphorylation; however, p-FAK decreased significantly when the two drugs were combined. In the past, many studies have confirmed that MMP-9 is involved in the invasion and metastasis of tumors and is related to the degree of malignancy and biological behavior of tumors (Ichim and Tait, 2016). The decrease in MMP-9 when two drugs were combined might contribute to the decreased migration ability of lung cancer cells in our experiment.

**Figure 9:**
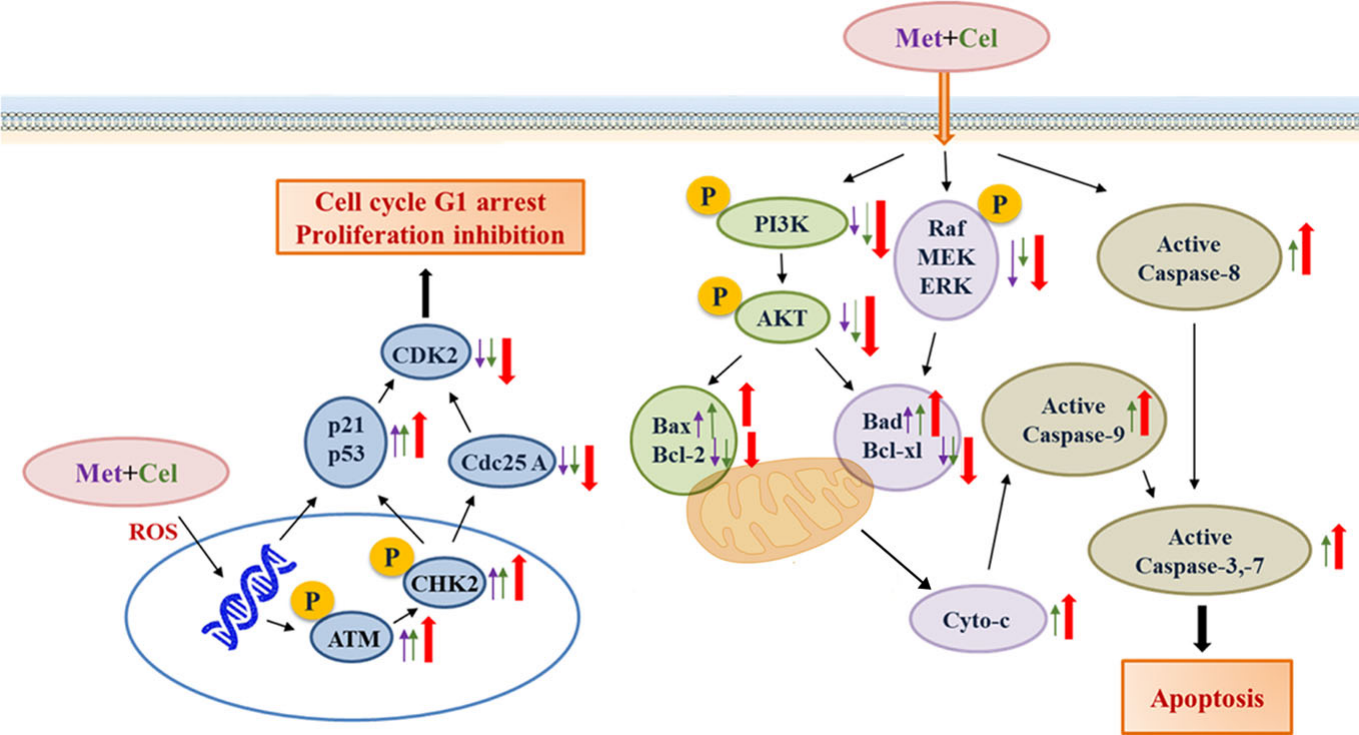
A working model for the synergistic effects of celecoxib and metformin on NSCLC cells. The purple arrow represents protein expression changes in response to metformin, the green arrow represents protein expression changes in response to celecoxib, and the red arrow represents protein expression changes in response to combination treatment with celecoxib and metformin.

In conclusion, celecoxib combined with metformin inhibits tumor growth by inhibiting cancer cell migration and proliferation, inducing cell cycle arrest, and promoting apoptosis. The activation of the p53 signaling pathway and the inhibition of RAS-Raf-ERK and PI3K/AKT, which are crucial for the survival of tumor cells, might be the reason why celecoxib combined with metformin exerts a good antitumor effect in NSCLC (**Figure 9**). Xenograft A549 tumor-bearing nude mice *in vivo* experiments strongly show the excellent effect of celecoxib combined with metformin in the treatment of NSCLC. Our data provide an effective combination of drugs for the treatment of NSCLC. Currently, since MEK or PI3K inhibitors fail to induce cancer cell death and lead to the selection of compensatory pathways that maintain tumor growth, we believe that celecoxib and metformin in combination can help overcome the resistance caused by crosstalk and feedback between cell pathways.

## Data Availability Statement

All datasets presented in this study are included in the article/supplementary material.

## Ethics Statement

The experiments were approved by Nanjing University Animal Care and Use Committee, and we strictly followed these rules during our experiments.

## Author Contributions

HZ and Z-CH designed the outline of the paper. HZ revised the manuscript. NC, YL, and JL contribute equally to this work. HZ, NC, and YL performed most experiments in this study. JL and YL wrote the manuscript and prepared the figures. YL and JL help with the cell related experiments. FC and XZ performed the experiments with the animals. HX and JC helped with the western bolt experiments. JL and YL provided thought-provoking discussion. All authors contributed to the article and approved the submitted version.

## Funding

This study was supported by grants from the Chinese National Natural Sciences Foundation (81773099, 81630092), the National Key R&D Program of China (2017YFA0506000).

## Conflict of Interest

Author Z-CH was employed by company Jiangsu TargetPharma Laboratories Inc.

The remaining authors declare that the research was conducted in the absence of any commercial or financial relationships that could be construed as a potential conflict of interest.
